# Efficient nested-PCR-based method development for detection and genotype identification of *Acanthamoeba* from a small volume of aquatic environmental sample

**DOI:** 10.1038/s41598-021-00968-2

**Published:** 2021-11-05

**Authors:** Tsui-Kang Hsu, Jung-Sheng Chen, Hsin-Chi Tsai, Chi-Wei Tao, Yu-Yin Yang, Ying-Chin Tseng, Yi-Jie Kuo, Dar-Der Ji, Jagat Rathod, Bing-Mu Hsu

**Affiliations:** 1 Department of Ophthalmology, Cheng Hsin General Hospital, Taipei, Taiwan; 2 School of Medicine, National Yang Ming Chiao Tung University, Hsinchu, Taiwan; 3 Department of Medical Research, E-Da Hospital, Kaohsiung, Taiwan; 4 Department of Earth and Environmental Sciences, National Chung Cheng University, No. 168, University Road, Minhsiung, Chiayi County, 621 Taiwan; 5 Department of Psychiatry, School of Medicine, Tzu Chi University, Hualien, Taiwan; 6 Department of Psychiatry, Tzu-Chi General Hospital, Hualien, Taiwan; 7 Section of Respiratory Therapy, Cheng Hsin General Hospital, Taipei, Taiwan; 8 Department of Laboratory, Show Chwan Memorial Hospital, Changhua, Taiwan; 9 Department of Orthopedic Surgery, Wan Fang Hospital, Taipei Medical University, Taipei, Taiwan; 10 Department of Tropical Medicine, National Yang Ming Chiao Tung University, Hsinchu, Taiwan; 11 Department of Earth Sciences, National Cheng Kung University, Tainan, Taiwan; 12 Center for Innovative on Aging Society (CIRAS), National Chung Cheng University, No. 168, University Road, Minhsiung, Chiayi County, 621 Taiwan

**Keywords:** Water microbiology, Environmental sciences

## Abstract

*Acanthamoeba* spp. are opportunistic human pathogens that cause granulomatous amoebic encephalitis and keratitis, and their accurate detection and enumeration in environmental samples is a challenge. In addition, information regarding the genotyping of *Acanthamoeba* spp. using various PCR methods is equally critical. Therefore, considering the diverse niches of habitats, it is necessary to develop an even more efficient genotyping method for *Acanthamoeba* spp. detection. This study improved the sensitivity of detection to avoid underestimation of *Acanthamoeba* spp. occurrence in aquatic environmental samples, and to accurately define the pathogenic risk by developing an efficient PCR method. In this study, a new nested genotyping method was established and compared with various PCR-based methods using in silico, lab, and empirical tests. The in silico test showed that many PCR-based methods could not successfully align specific genotypes of *Acanthamoeba*, except for the newly designed nested PCR and real-time PCR method. Furthermore, 52 water samples from rivers, reservoirs, and a river basin in Taiwan were analysed by six different PCR methods and compared for genotyping and detection efficiency of *Acanthamoeba*. The newly developed nested-PCR-based method of genotyping was found to be significantly sensitive as it could effectively detect the occurrence of *Acanthamoeba* spp*.*, which was underestimated by the JDP-PCR method. Additionally, the present results are consistent with previous studies indicating that the high prevalence of *Acanthamoeba* in the aquatic environment of Taiwan is attributed to the commonly found T4 genotype. Ultimately, we report the development of a small volume procedure, which is a combination of recent genotyping PCR and conventional real-time PCR for enumeration of aquatic *Acanthamoeba* and acquirement of biologically meaningful genotyping information. We anticipate that the newly developed detection method will contribute to the precise estimation, evaluation, and reduction of the contamination risk of pathogenic *Acanthamoeba* spp., which is regularly found in the water resources utilised for domestic purposes.

## Introduction

*Acanthamoeba* is the most common type of free-living amoeba that occurs in different environments related to human diseases^1,2^. It has been isolated from diverse domestic and natural environments, such as freshwater lakes, swimming pools, marine water, drinking water, contact lens washing solutions, ventilation systems, dialysis equipment, and soil among others^2–8^. *Acanthamoeba* can cause an infection in the central nervous system (CNS) called granulomatosis amoebic encephalitis (GAE), as well as lung and skin infections^9^. Moreover, *Acanthamoeba* can cause *Acanthamoeba* keratitis (AK), an infection of the cornea that may lead to different levels of vision loss^10–12^. This infection is becoming more common in industrialised countries because of the increased use of contact lenses and poor handling habits, which are some of the risk factors associated with its pathology^13^. In addition, multiple studies revealed that *Acanthamoeba* is prevalent in spring water, entertainment parks, and swimming pools. Hence, it is important to monitor the presence of *Acanthamoeba* in aquatic environments^2,13–15^.

Twenty four species have been identified in the genus *Acanthamoeba* based on their morphology. Species identification based on morphology was considered unreliable because culture conditions effect cyst morphology^16^; thus, more advanced methods were considered necessary to name different species precisely. Originally, a genotype in *Acanthamoeba* was arbitrarily defined as including all strains whose 18S rRNA gene sequences exhibited less than 6% divergence from one another in a standard sequence alignment; this criterion was later adjusted to divergences less than 5%^17^. In *Acanthamoeba* species, genotypes are referred to as ’T-types’, which are designated from T1-T21^2,18^. The most common pathogenic genotype of *Acanthamoeba* in soft contact lens users and the environment is T4, with an occurrence of approximately 75–80%. It is also the main causative agent of GAE, AK, and other infections; more than 94% of keratitis cases were found to be linked with this genotype. Moreover, T4 exhibits significantly high binding to host cells and causes severe cytotoxicity compared to other genotypes. The other genotypes of pathogenic *Acanthamoeba* include T1, T2, T10, and T11, etc.^19^.

Microscopic examination with culture method has traditionally been regarded as the gold standard for *Acanthamoeba* diagnosis in the past. Advanced molecular approaches focusing on 18S rRNA genes are currently the gold standard for genotyping characterisation of *Acanthamoeba* species^20,21^. JDP-PCR is the most common clinical diagnosis method used to determine the genotype of *Acanthamoeba*, but testing methods in water are more diverse; however, each method has its advantages and disadvantages. Quantitative PCR (qPCR) is the preferred method of detection because of its increased sensitivity^13,22^, but its inability to determine the genotype, which is associated with pathogenicity, is identified as a potential drawback of this method. In addition to using qPCR, a study by Magnet et al. 2013 showed the presence of *Acanthamoeba* DNA in 211 of the 223 water samples; however, only 39 qPCR positive samples were amplified by the JDP-PCR typing method^13^. It is generally believed that the sensitivity of qPCR is better than that of general one-step JDP-PCR. Therefore, the classification and occurrence of *Acanthamoeba* in environmental water samples is controversial and may be attributed to the limited sensitivity of JDP-PCR. Several studies in the past regarding genotyping methods for aquatic *Acanthamoeba* have indicated that the culture method combined with JDP-PCR is more sensitive, in contrast to a few studies, which indicate the direct concentration from water bodies with JDP-PCR to be more sensitive. However, many studies have also suggested that the use of a combination of different methods may have higher sensitivity^13,23,24^.

In several studies, nested PCR which involves a combination of different PCRs, has emerged as a successful technique for detecting protozoan/fungal pathogens in water samples^25–28^. Nested PCR is more sensitive than one-step PCR and equally or more sensitive than qPCR^29,30^. Recent studies have developed new nanoparticle-assisted PCRs for detection of *Acanthamoeba*^31,32^. Due to the emergence of a new genotype in recent years, no study has been carried out to investigate the detection ability of primers currently being used for *Acanthamoeba* detection^33^. Although semi-nested and nested PCR methods have advantages such as double the detection rate of the original one-step JDP-PCR, they have some limitations. For example, the size of the PCR product is too small, and the primers are unable to align to some genotypes of *Acanthamoeba*, including T7, T8, T9, T17, and T18^4,23^. The JDP genotyping primers for *Acanthamoeba* detection, as described by Schroeder et al. 2001 were a part of the amplified product (1000 bp) with the common free-living amoebae (FLA) 18S primer set that included the DNA of *Acanthamoeba* as described by Coskun et al.^34,35^. We hypothesised that the use of common FLA primer set as outer PCR in conjunction with the use of a JDP primer set as inner PCR in a nested PCR reaction can effectively enhance sensitivity and can be used for genotyping. To confirm the specificity of the nested primer set, the primer sequences were aligned with sequences from all genotypes of *Acanthamoeba* using the Molecular Evolutionary Genetics Analysis (MEGA) software (http://www.megasoftware.net). The results showed that the common FLA primer set could align with all the genotypes, but the JDP1 primer could not align with genotypes T9, T17, and T18; JDP2 could not align with genotypes T7, T8, T9, T17, and T18. However, the forward primer (AcanF900) used in qPCR could align with all the genotypes and was identical to JDP1^36^ with the only difference being the absence of the last two nucleotides in the 5’ region. To circumvent this limitation, the nucleotide at position 15 was changed from A to R (A/G) in the JDP2 primer, which optimally modified it (JDP2-M) and allowed it to align with all the genotypes. Hence, we hypothesise that the use of Optimally Modified nested PCR (common FLA primer set combined with an inner primer set, AcanF900 + JDP2-M) can effectively detect all genotypes of *Acanthamoeba* with high sensitivity. Therefore, the purpose of this study was to compare the limitations of the currently available PCR methods used to detect *Acanthamoeba*, and study the relationship between sample concentration and proliferation process on various environmental surface water bodies. Six PCR-based methods used to detect the presence of *Acanthamoeba* in environmental water and their limitations for in situ applications were compared. We hypothesise that the use of the Optimally Modified Genotyping Nested PCR method will enhance the detection limit and result in the requirement of less than one liter of water sample for the detection of *Acanthamoeba* in aquatic environments. The other aim of this study was to use this combinatorial primer approach in qPCR to provide necessary information about the quantity and genotypes of *Acanthamoeba* in aquatic environments.

## Materials and methods

### PCR methods for *Acanthamoeba*

Six PCR based methods used in this study to detect *Acanthamoeba*, were Genotyping PCR (M1), Genotyping Nested PCR (M2), Optimal Modified Genotyping Nested PCR (M3), Scheikl Genotyping Nested PCR (M4), Genotyping Semi-nested PCR (M5), and Qvarnstrom Real-time PCR (M6), respectively. Primer sequences used for each PCR method are given in Table 1.

**Table 1:** The details of primers used by different PCR methods and their genotype detection limitation results for *Acanthamoeba* spp. by in Silico analysis.

Methods	Primers	Sequence (5′ → 3′)	Length (bp)	Un-detected genotype	References
JDP-genotyping PCR (M1)	JDP 1	GGC CCA GAT CGT TTA CCG TGA A	440–550	T9, T17, T18	Schroeder et al.^34^
	JDP 2	TCT CAC AAG CTG CTA GGG GAG TCA		T7, T8, T9, T17, T18	
Optimal modified genotyping nested PCR (M3)	ComFLA F (outer)	CGC GGT AAT TCC AGC TCC AAT AGC	980–1090	Nil	Coskun et al.^35^
	ComFLA R (outer)	CAG GTT AAG GTC TCG TTC GTT AAC		Nil	
	AcanF900 (inner)	CCC AGA TCG TTT ACC GTG AA	440–550	Nil	This study
	JDP2-M (inner)	TCT CAC AAG CTG CTR GGG GAG TCA		Nil	
Genotyping nested PCR (M2)	ComFLA F (outer)	CGC GGT AAT TCC AGC TCC AAT AGC	980–1090	Nil	Coskun et al.^35^
	ComFLA R (outer)	CAG GTT AAG GTC TCG TTC GTT AAC		Nil	
	JDP 1 (inner)	GGC CCA GAT CGT TTA CCG TGA A	440–550	T9, T17, T18	Schroeder et al.^34^
	JDP 2 (inner)	TCT CAC AAG CTG CTA GGG GAG TCA		T7, T8, T9, T17, T18	
Scheikl genotyping nested PCR (M4)	JDP 1 (outer)	GGC CCA GAT CGT TTA CCG TGA A	920–1030	T9, T17, T18	Scheikl et al.^4^
	P3rev (outer)	CTA AGG GCA TCA CAG ACC TG		Nil	
	P2fw (inner)	GAT CAG ATA CCG TCG TAG TC	120–160	T7, T8, T9, T17, T18	
	JDP 2 (inner)	TCT CAC AAG CTG CTA GGG GAG TCA		T7, T8, T9, T17, T18	
Semi-nested PCR (M5)	JDP 1 (outer)	GGC CCA GAT CGT TTA CCG TGA A	440–550	T9, T17, T18	Dhivya et al.^23^
	JDP 2 (outer)	TCT CAC AAG CTG CTA GGG GAG TCA		T7, T8, T9, T17, T18	
	A1 (inner)	AAC GAT GCC GAC CAG CGA TTA	120–160	T7, T8, T9, T17, T18	
	JDP 2 (inner)	TCT CAC AAG CTG CTA GGG GAG TCA		T7, T8, T9, T17, T18	
Real-time PCR (M6)	AcanF900	CCC AGA TCG TTT ACC GTG AA	180	Nil	Qvarnstrom et al.^36^
	AcanP1000	FAM—CTG CCA CCG AAT ACA TTA GCA TGG—BHQ1		Nil	
	AcanR1100	TAA ATA TTA ATG CCC CCA ACT ATC C		Nil	

*M1 (also called JDP PCR)* Initially, the primers used for the polymerase chain reaction to detect *Acanthamoeba* spp. were JDP1 and JDP2^34^. These primers can be used to amplify 18S rRNA gene sequence of ASA.S1 variant segment in *Acanthamoeba*, polymerase chain reaction using this primer set is named JDP Genotyping PCR. The reaction mixture was prepared as follows: 1 μL of 10 μM forward primer, 1 μL of 10 μM reverse primer, 5 μL of Fast-Run™ Taq Master Mix with Dye (Protech) and 3 μL of DNA template and sterile water to make up the volume to 25 μL. The PCR reaction conditions consist of three steps: (1) Preliminary denaturation: 95 °C/5 min, (2) 35 Cycles of 95 °C/15 s, 62 °C/15 s, 72 °C/30 s. (3) extension at 72 °C/10 min.

*M2* Due to the limitation of detection limit, Genotyping PCR performed by JDP1 and JDP2 primers, a nested polymerase chain reaction was used to amplify the ASA.S1 variant of the 18S rRNA gene sequence in *Acanthamoeba* ribosome, and hence this nested polymerase chain reaction was named as Genotyping Nested PCR. The outer primer set used in the first PCR included ComFLA-F and ComFLA-R^35^, and the inner primer set used for the second PCR included JDP1 and JDP2. The first-step reaction mixture was prepared by mixing 1 μL of 10 μM forward primer, 1 μL 10 μM reverse primer, 5 μL Fast-Run ™ Taq Master Mix with Dye (Protech) and 5 μL DNA template and the volume of the reaction was made up to 25 μL with sterile water. The PCR reaction conditions included three steps: (1) Preliminary denaturation: 94 °C/7 min, (2) 45 cycles of 94 °C/1 min, 60 °C/1 min, 72 °C/1 min, (3) extension at 72 °C/10 min. The second-step reaction mixture was prepared by mixing 1 μL of 10 μM forward primer, 1 μL of 10 μM reverse primer, 5 μL of Fast-Run™ Taq Master Mix with Dye (Protech) and 1 μL of the first-step PCR product and the final volume was made up to 25 μL with sterile water. The PCR reaction conditions included three steps: (1) Preliminary denaturation: 95 °C/5 min, (2) 35 cycles of 95 °C/15 s, 62 °C/15 s, 72 °C/30 s, (3) extension at 72 °C/10 min.

*M3* Although the limitation of detection limit is overcome, the JDP1 and JDP2 primers still cannot detect some genotypes. Therefore, a series of primers AcanF900 and JDP2-M designed in this study were used in a nested polymerase chain reaction, to effectively amplify the ASA.S1 variant of *Acanthamoeba* of all genotypes and it was named as M3. The outer primer set used in the first PCR included ComFLA F and ComFLA R, and the inner primer set used in the second PCR included AcanF900 and JDP2-M. The thermal cycling conditions used for M3 are the same that were used for Genotyping Nested PCR.

*M4* In addition to the above-mentioned three PCR methods, this study also included the use of the nested polymerase chain reaction method published by Scheikl et al. 2014, and directly named this method as Nested PCR. The outer primer set used in the first PCR included JDP1 and P3rev, and the inner primer set used in the second PCR included P2fw and JDP2^4^.

*M5* Our study also includes the use of the semi-nested polymerase chain reaction method published by Dhivya et al. (2007) and named this method ‘Semi-nested PCR’. The outer primer set used in the first PCR included JDP1 and JDP2, and the inner primer set used in the second PCR included A1 and JDP2^23^.

*M6* Qvarnstrom et al. (2006) published a quantitative real-time polymerase chain reaction method to quantify the concentration of protozoa in water. The primers used for this qPCR are AcanF900 and AcanR1100, and the probe is AcanP1000. The reaction mixture was prepared as follows: 0.8 μL of 10 μM forward primer, 0.8 μL of 10 μM reverse primer, 0.8 μL probe at 10 μM AcanP1000, 10 μL EZtime™ Fast Reverse-Time PCR 2 × Premix for TaqMan^®^ Probe and 3 μL of DNA template and sterile water to make up the final volume to 20 μL. The PCR reaction conditions included two steps: (A) Preliminary denaturation: 95 °C/20 s. (B) 45 cycles of 95 °C/3 s, 58 °C/30 s^36^. This method is considered as the gold standard for *Acanthamoeba* detection^36^.

### Construction of positive control

The positive control DNAs were from our previous study, environmental *Acanthamoeba* strain (T4 genotype) was isolated from Taiwan and standard positive ATCC 30010 (As a gift from NCKU, Taiwan) specimens^8^. The Extracted DNA from the isolated strain was then amplified by a one-step PCR approach using a specific ComFLA primer set. Subsequently, PCR products were cloned into T&A Cloning Vector (Reverse Biotech Co., Taiwan) and transformed into JM109 competent *Escherichia coli* cells. After selection of clones and verification of the inserts, plasmids were extracted using Gene-Spin™ MiniPrep Plasmid Purification Kit (Protech, Taiwan) according to the manufacturer’s instructions. The extracted plasmids were used as positive controls. DNA quality and quantity were estimated by a NanoDrop spectrophotometer.

### Evaluation of detection limit

To determine the limit of detection (LOD) for each method (excluding M6 method), a 13-fold serial dilution of plasmid DNA was prepared. This created 5 × 10^−2^ to 5 × 10^10^ plasmid copies per reaction. After the PCR was completed, samples were checked through electrophoresis on a 2% agarose gel and the DNA products were visualized using a UV transilluminator. The amplified PCR products of diluted samples were checked in each lane of the gel to determine the limit of detection.

### Sample collection

**Figure 1:**
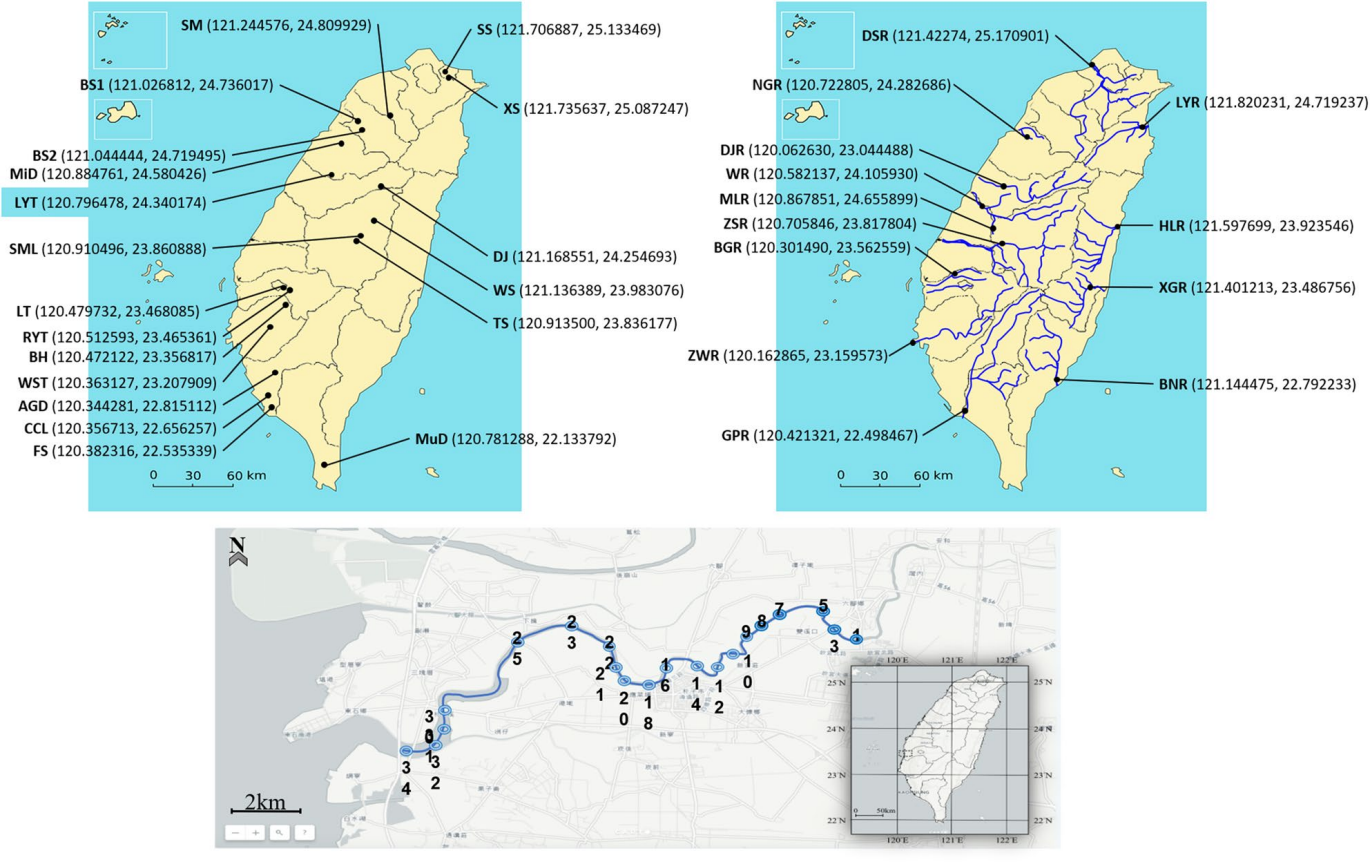
Sampling locations of the 14 freshwater reservoirs, 11 major rivers and Puzhi river basin in Taiwan. The figure (left) is reservoir locations and figure (right) is river’s locations, whereas figure (down) is Puzhi river basin. The approximate geographical coordinates (latitude/longitude) of sampling site were attached after each sample name. The Fig. 1 is modified from free-download website and this image is searched from cc0 search website (http://cc0.wfublog.com) that is under the CC-0 license (https://goo.gl/fLmlHJ).

Water samples were collected from 13 rivers and 19 freshwater reservoir locations in Taiwan as described in our previous study (Fig. 1)^37^. The rivers were located in the four cardinal regions and the freshwater reservoirs were located in the northern, central, and southern regions in Taiwan (Detailed coordinates of each sampling site are shown in Fig. 1). The selected total 19 freshwater reservoirs and 9 of the total 13 rivers i.e. DSR, NGR, HLR, DJR, ZSR, ZWR, BNR, WR, and LYR were the principal sources of drinking water in Taiwan. The part of 7 rivers shed i.e. DSR, XGR, LYR, ZSR, NKR, ZWR and GPR and 8 reservoirs i.e. SM, SML, LYT, LT, RYT, WST, CCL, and AGD were the recreational areas or Waterfront Park for shipping or recreational activities. Sample collections were carried out in the summer of 2016. In addition, we have added the Puzih River basin (23° 28′ N, 120° 13′ E) survey. We carried out *Acanthamoeba* detection from the Puzih River basin (23° 28′ N, 120° 13′ E) and all sampling sites are the same as our previous study (Fig. 1)^38^. One-liter samples were taken from the water surface at each of the 32 rivers and freshwater reservoirs locations. The samples were stored at ambient temperature and analyzed within 8 h of sample collection. Each water sample was concentrated and then used for detection of *Acanthamoeba* by several PCR-based methods, cloning and sequencing analysis.

### Empirical test of aquatic environmental *Acanthamoeba* by six PCR-based methods

In this study, the *Acanthamoeba* was detected from environmental water in Taiwan by direct concentration procedure and non-nutrient agar (NNA) and liquid state (PAS) culture procedure (Culture procedure).

1. To detect *Acanthamoeba,* 1 L water sample was collected*,* concentrated by filtration, and subjected to DNA extraction to obtain genomic DNA. Detailed procedure the Direct concentration method is described in our previous study^39^.
2. A total of 300 mL environmental water sample was filtered, and the filter was stuck to non-nutrient agar (NNA) with smeared *Escherichia coli* in the outer circle. The morphology of free-living amoebae on NNA medium was observed by using a high-power inverted microscope and label the suspected amoeba on the medium to extract DNA after purification. Refer to our previous study for the Culture method procedure^39^.
3. The obtained DNA was qualitatively confirmed by each PCR-based method. Refer to 2.1 of this study for the detection and genotyping procedure.

### Sequencing and identification of *Acanthamoeba*

The PCR products generated from M3 were used for denaturing gradient gel electrophoresis (DGGE) analysis as described in our previous study^8^. PCR products were electrophoresed on 2% agarose gel (Biobasic Inc., Canada), stained with a solution of ethidium bromide and visualized under UV light. All positive PCR products were further cloned by T&A Cloning kit (Real Biotech Co., Taiwan). Cloning was performed by ligating the PCR product in T&A Cloning Vector and transforming it into *E. coli* DH5α cells. For each cloned sample, approximately three colonies were selected for PCR confirmation. Plasmid DNA was subsequently extracted from the confirmed colonies by the Plasmid DNA Extraction kit according to the manufacturer’s instructions for sequencing. All positive amplicons were sequenced under Applied Biosystems 3730xl DNA Analyzer by Mission Biotech Taiwan. All nucleotide sequences were assessed in National Center for Biotechnology Information (NCBI) GenBank database using the PubMed NCBI BLAST program for genotype confirmation. All sequence data from the samples have been submitted to GenBank (at www.ncbi.nlm.nih.gov) and the assigned accession numbers were from MK390840- MK390877.

### Statistical analysis

The sensitivity, specificity and accuracy of each assay were calculated using the following formulas by comparison with a “gold standard (M6)” and by comparison with sequencing outcome:

1. $${\text{Sensitivity}} = {\text{TP/}}\left( {{\text{TP}} + {\text{FN}}} \right) = \left( {{\text{Number}}\;{\text{of}}\;{\text{true}}\;{\text{positive}}\;{\text{assessment}}} \right){/}\left( {{\text{Number}}\;{\text{of}}\;{\text{all}}\;{\text{positive}}\;{\text{assessment}}} \right)$$
2. $${\text{Specificity}} = {\text{TN/}}\left( {{\text{TN}} + {\text{FP}}} \right) = \left( {{\text{Number}}\;{\text{of}}\;{\text{true}}\;{\text{negative}}\;{\text{assessment}}} \right){/}\left( {{\text{Number}}\;{\text{of}}\;{\text{all}}\;{\text{negative}}\;{\text{assessment}}} \right)$$
3. $${\text{Accuracy}} = \left( {{\text{TN}} + {\text{TP}}} \right){/}\left( {{\text{TN}} + {\text{TP}} + {\text{FN}} + {\text{FP}}} \right) = \left( {{\text{Number}}\;{\text{of}}\;{\text{correct}}\;{\text{assessments}}} \right){\text{/(Number}}\;{\text{of}}\;{\text{all}}\;{\text{assessments}})$$

### Ethical standards

The manuscript does not contain clinical studies or patient data. The authors declare that they have no conflict of interest.

## Results and discussion

### Evaluation of the suitability of various primers for each PCR-based method for detection of genotype *Acanthamoeba*

Analysis of the 18S rRNA gene interval for *Acanthamoeba* spp. showed that the location of the common FLA 18S primer set (ComFLA F and ComFLA R) was just in the outer part of the region covered by JDP primers. Therefore, we designed the common FLA 18S primer set as the outer PCR primers, and used JDP primer set as the inner PCR primers. We termed this method as Genotyping Nested PCR. 18S rRNA gene sequences of genotype T1 to T20 were collected from the NCBI database and were subjected to BLAST analysis. It was noted that the JDP primer set could not successfully align to some sequences of the genotypes, such as forward primer JDP 1 excluded the genotypes T9, T17 and T18, while the reverse primer excluded the genotypes T7, T8, T9, T17 and T18. These mismatches between primers and genotype sequences may lead to the failure of JDP-PCR-based detection of these types of *Acanthamoeba*. Therefore, we designed a set of primers, AcanF900 and JDP2-M, as the inner primers for nested PCR to successfully amplify the ASA.S1 mutated segment of 18S rRNA gene sequence in all genotypes of *Acanthamoeba*. We termed this method as Optimal Modify Genotyping Nested PCR. In this method, the outer primer set used in the outer PCR included ComFLA F and ComFLA R, and the inner primer set used in the second PCR included AcanF900 and JDP2-M.

The present study includes a comparative analysis of our self-designed nested PCR with the nested PCR method by Scheikl et al. (2014) and the semi-nested PCR method by Dhivya et al. (2007). BLAST analysis showed no alignment of the sequences of primers P2fw and A1 from Scheikl nested PCR and Dhivya semi-nested PCR with the genotypes T7, T8, T9, T17 and T18. Therefore, these types of environmental *Acanthamoeba* may not be detected by these two PCR methods. The primers and probe of M6 could align to all genotypes of *Acanthamoeba*. Details of primers used for different methods of PCR detection for each *Acanthamoeba* spp. including primer sequences, length of PCR products (bp) and un-detected genotypes are shown in Table 1. Further, we determined the LOD of each PCR-based method for detecting *Acanthamoeba* by serial dilution assay.

### Testing of the methods

**Figure 2:**
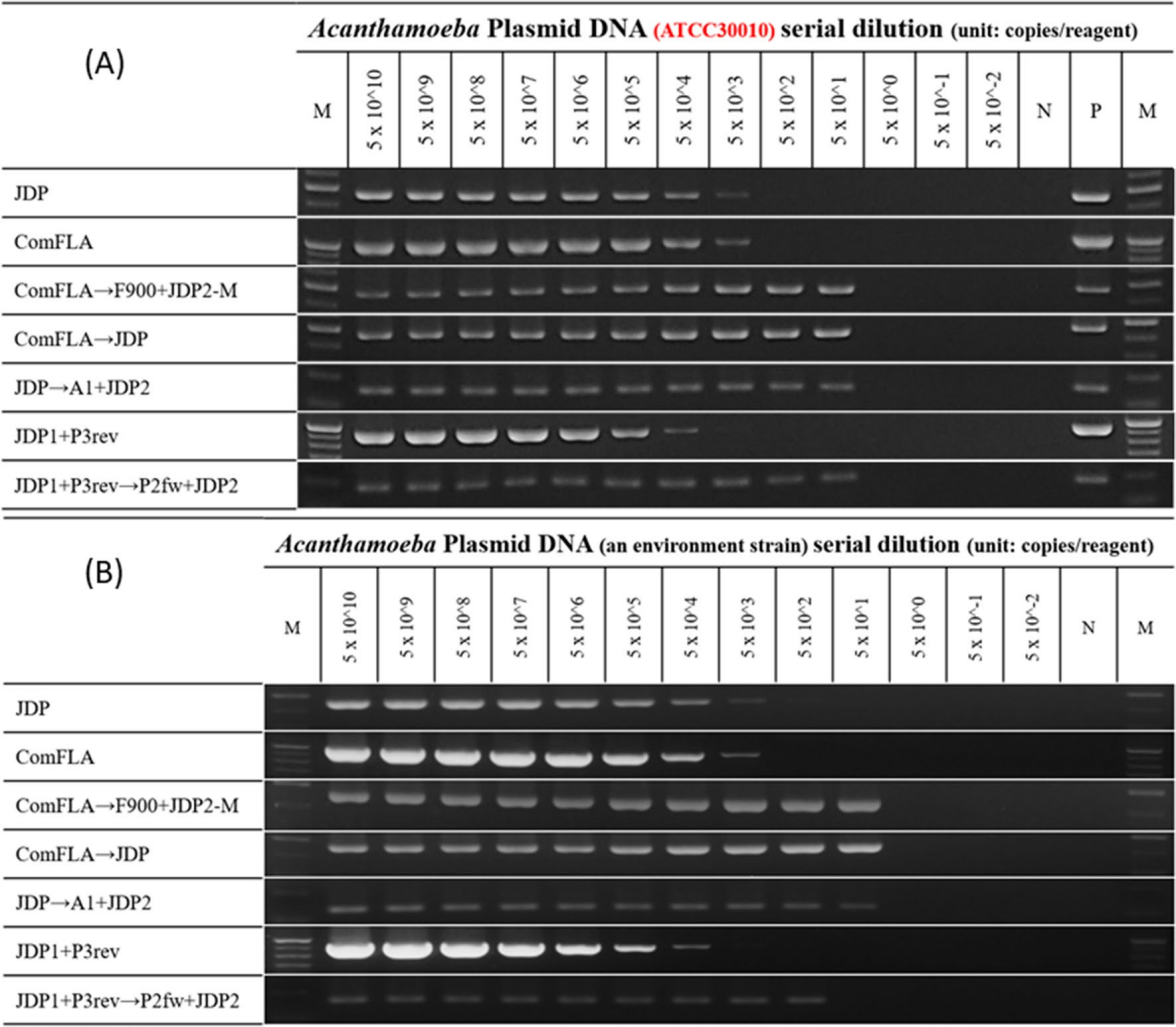
The limit of detection (LOD) of *Acanthamoeba* spp. by various PCRs. The positive control of figure (A) is ATCC30010, whereas the positive control of figure (B) is an environmental strain from Taiwan. The M and N indicated the 100 bp-Marker and the negative control, respectively. The copy number per reaction is shown at the top of each lane. All the PCR amplicon results of Fig. 2 A and B have been placed in website Figshare (https://figshare.com/s/ff4fa72321b08b3e86d9).

The LOD for *Acanthamoeba* was 5 × 10^4^ copies/reaction when analysed using the one-step outer (JDP1 + P3rev) PCR method based on semi-nested PCR (~ 930 bp), whereas it was 5 × 10^3^ copies/reaction when analysed by the one-step JDP (~ 450 bp) and ComFLA PCR method (~ 990 bp) (Fig. 2A and B). In the case of the nested PCR method described by Scheikl et al. (2014) that used a combination of the outer (JDP1 + P3rev) and inner (P2fw + JDP2) PCR methods, the LOD was decreased to 5 × 10^1^ and 5 × 10^2^ copies/reaction in two positive control assays. A further decrease in the LOD (5 × 10^1^ copies/reaction) was noted in the case of one semi-nested (a combination of JDP PCR and inner (A1 + JDP2) PCR methods) and two nested PCR assay methods—a combination of ComFLA and JDP PCR, and a combination of ComFLA and optimal modified JDP PCR methods (A1 + M-JDP2).

The detection limit of JDP Genotyping PCR was about 5000 copies. PCR results of genomic DNA from environmental samples were even more confusing indicating lower efficacy of detection due to limited template-primer binding. Thus, JDP Genotyping PCR when used for detection from environmental samples, had a worse detection limit (over 5000 copies/reaction). ComFLA were the first PCR primer sets of M3 and Genotyping Nested PCR. The detection limit of ComFLA PCR, 5000 copies, was similar to that of JDP PCR. From the results of second-step PCR, it was noted that the two types of nested PCR designed in this experiment successfully reduced the detection limit to 50 copies. Brighter bands observed after electrophoresis were confirmed the presence of several original DNA amplicons at a concentration of 50 (copies/reaction). Unlike the first-step PCR product, the concentration of the second-step PCR product did not decrease with the initial concentration of the DNA template. The concentration of the original DNA template tested in the electrophoresis 5 × 0^10^ (copies/reaction) to 5 × 10^1^ (copies/reaction) showed several nucleic acid products (brighter bands), as the amplified target DNA sequence from first-step PCR might have amplified to a higher extent in the second step as shown in Fig. 2. This finding implied that the real LOD of M3 was between 5 × 10^1^ (copies/reaction) and 5 × 10^0^ (copies/reaction). However, second-step PCR is needed to easily examine the amplification of the target sequence by gel electrophoresis.

LOD for semi-nested PCR method determined in this study i.e. 50 copies/reaction matches with that of the original study^23^. However, due to the shorter length of PCR products, ranging from 120 to 160 bp, bands with lighter intensity were observed on gel electrophoresis as compared to the M3. Moreover, the LOD for first-step and second-step M4 were approximately 5000 copies and 500 copies, respectively. M4 reduced only one order of the detection limit. The unremarkable DNA amplification with the semi-nested PCR method was due to the same reason. The large difference between the product size of first-step PCR (1000 bp) and second-step PCR (100 bp) might be responsible for the poor LOD of M4. Moreover, previous studies have reported successful use of the LAMP technique (loop-mediated isothermal amplification) for the detection of *Acanthamoeba* spp. from clinical and environmental samples with comparable performance with M6^40–42^. Chang et al. demonstrated the significant contribution of M6 towards a better understanding of the distribution and abundance of *Acanthamoeba* in an environment^5^. Previous studies have reported the LOD for M6 as approximately 55 copies/reaction or 0.1 fg/µL^43^. Therefore, we considered that the LOD for M3 provided similar sensitivity with real-time PCR (included M6) or LAMP, whereas our method provided better resolution in agarose gel electrophoresis and advantages in genotyping. The occurrence of environmental *Acanthamoeba* may have been underestimated by one-step JDP PCR. Therefore, our results suggest that the M3 will provide higher sensitivity for detecting environmental *Acanthamoeba*. With these promising results, we carried out the empirical tests of each PCR-based method in aquatic environments (Genotyping Nested PCR method excluded). Many environmental studies that include testing of microorganisms often use nested PCR to enhance the efficiency of studies by requiring small volume sample collection^26–28,37^; thus we hypothesize that M3 combined with small water sample collection will be useful for detecting *Acanthamoeba*.

### Sensitivity, specificity and accuracy of each PCR-based method for *Acanthamoeba* detection in water samples

A total of 32 surface water samples processed with direct concentration and Culture pretreatment procedure were analyzed by each PCR-based method. In comparison with the gold standard method (M6), the sensitivity, specificity and accuracy of other PCR-based methods were determined, as described in Table 2. For all the tested methods, with the combination of direct concentration and culture pretreatment procedure, the sensitivity of *Acanthamoeba* detection in water samples ranges from 54 to 100%. Among all the PCR methods, the M3 and M5 showed the highest sensitivity in the direct concentration procedure, culture procedure and under total detection circumstances. The single-step Genotyping PCR (M1) showed the poor sensitivity of detection compared to other methods, it is not agreed with the clinical study^21^ The difference may be due to the concentration of the sample, clinical samples were higher than environmental samples. The specificity was presented a different result compared to sensitivity, the highest specificity was shown in M1. However, it is caused by the efficiency of the gold standard method (M6). In other words, the gold standard method (M6) is not the most powerful approach for *Acanthamoeba* detection. Therefore, we showed the sensitivity, specificity and accuracy of each PCR-based method by identifying the positive sequencing outcome in Supplementary Table 1. The sensitivity and accuracy of M6 were 86% and 88% in total detection circumstances, respectively. Furthermore, the M3 method was shown the best sensitivity (96%) and accuracy (97%) than other methods, and M1 method also was shown poor sensitivity (32 to 50%) and accuracy (41 to 56%) in whichever pretreatment process. These findings indicate that many studies involving the detection of environmental *Acanthamoeba* underestimated the detection rate by using single-step Genotyping (JDP) PCR^6,16,34,39,44,45^. Many environmental microbiology studies have proven the sensitivity of nested PCR and real-time PCR for the determination of the actual detection rate in the environment^29,30^. A previous study evaluated two currently available real-time PCR methods for the detection of *Acanthamoeba* spp. showed better sensitivity of detection by M6 as compared to Riviere Real-time PCR^5^. In line with our results, the same study showed poor diagnostic and analytic sensitivity of PCR using the JDP primer set or F900-R100 primer set (50–53.6%) as compared to M6 (82.1–89.3%)^5^. However, the results of this empirical test showed better sensitivity of detection with the nested PCR and semi-nested PCR as compared to M6, except M4. The poor LOD (5 × 10^2^ copies/reaction) of M4 may have been responsible for its poor sensitivity. Furthermore, our study justifies the two studies on aquatic environmental *Acanthamoeba* in Spain that showed high detection rates of *Acanthamoeba* in water samples (over 90%) by real-time PCR, and poor genotyping results by Genotyping (JDP) PCR in *Acanthamoeba* positive samples^13^. In the present study, the sensitivity of the M3 method based on the positive control test and the empirical test was found better than that with either of the PCR-based methods, which is in agreement with our initial hypothesis.

**Table 2:** Methods comparison for calculating sensitivity from empirical test based on Qvarnstrom real-time PCR-positive sample (as gold standard method).

Methods	Sensitivity			Specificity			Accuracy		
	D.C	Culture	Total	D.C	Culture	Total	D.C	Culture	Total
Genotyping PCR (M1)	52% (11/21)	62% (8/13)	54% (13/24)	91% (10/11)	95% (18/19)	85% (7/8)	66% (21/32)	81% (26/32)	63% (20/32)
Optimal modified genotyping nested PCR (M3)	95% (20/21)	100% (13/13)	96% (23/24)	64% (7/11)	95% (18/19)	50% (4/8)	84% (27/32)	97% (31/32)	84% (27/32)
Scheikl genotyping nested PCR (M4)	74% (17/23)	85% (11/13)	75% (18/24)	64% (7/11)	95% (18/19)	50% (4/8)	75% (24/32)	91% (29/32)	69% (22/32)
Genotyping semi-nested PCR (M5)	95% (20/21)	100% (13/13)	96% (23/24)	73% (8/11)	95% (18/19)	63% (5/8)	88% (28/32)	97% (31/32)	88% (28/32)

### Empirical test of *Acanthamoeba* in rivers and reservoirs by each PCR-based method

The *Acanthamoeba* detection results by various PCR-based methods combined with direct concentration and Culture pretreatment procedure are summarized in Table 3. Among the 32 samples analyzed, *Acanthamoeba* was detected in 28 samples (87.5%), making the detection rate ranging from 28.1% to 75% based on different methods. The presence of *Acanthamoeba* was highest in 24 samples (75%) tested using M3 combined with direct concentration methods and in 9 samples (28.1%) tested using Genotyping (JDP) PCR combined Culture method. The amplicons from various PCR-based methods were sequenced to determine the genotypes excluding the M6. Among the total 28 *Acanthamoeba* positive water samples, the most predominant genotype was T4 (21/28, 75%). T2 (4/28, 14.3%), T3 (2/28, 7.1%), T5 (1/28, 3.6%) and T11 (1/28, 3.6%) genotypes were also found. One of the *Acanthamoeba* positive water sample from Zengwen was positive for both T5 and T4 genotypes as confirmed by the DGGE assay. *Acanthamoeba* spp. concentrations in rivers and reservoirs samples were in the range of 7.2 × 10^2^–3.8 × 10^7^ copies/L as determined by M6.

**Table 3:** Summary of different PCR methods used to detect *Acanthamoeba* in the aquatic environmental samples.

Sampling locations	Genotyping PCR (M1)		Modify genotyping nested PCR (M3)		Nested PCR (M4)		Semi-nested PCR (M5)		Real-time PCR (M6)	
	JDP		ComFLA → F900 + JDP2-M		JDP1 + P3rev → P2fw + JDP2		JDP → A1 + JDP2		AcanF900 + AcanP1000 + AcanR1100	
	D.C	Culture	D.C	Culture	D.C	Culture	D.C	Culture	D.C. (copies/L)	Culture
DSR	+ (T4)	+ (T4)	+ (T4)	+ (T4)	+ (T4)	+ (T4)	+ (T4)	+ (T4)	+_(1348)_	+
LYR	–	–	+ (T4)	–	–	–	+ (T4)	–	+_(3924)_	–
XGR	+ (T4)	–	+ (T4)	–	+ (T4)	–	+ (T4)	–	+_(1527)_	–
HLR	–	–	+ (T4)	–	+ (T4)	–	+ (T4)	–	+_(751)_	–
XS	–	–	–	–	–	–	–	–	–	–
SS	+ (T4)	+ (T4)	+ (T4)	+ (T4)	+ (T4)	+ (T4)	+ (T4)	+ (T4)	+_(7100)_	+
SM	–	–	+ (T4)	–	–	–	+ (T4)	–	+_(2563)_	–
BS1	+ (T2)	–	+ (T2)	+ (T2)	+ (T2)	+ (T2)	+ (T2)	+ (T2)	+_(99,362)_	+
BS2	+ (T4)	+ (T4)	+ (T4)	+ (T4)	+ (T4)	+ (T4)	+ (T4)	+ (T4)	+_(477,527)_	+
BGR	–	–	+ (T4)	–	+ (T4)	–	+ (T4)	–	–	–
WR	+ (T2)	–	+ (T2)	–	+ (T2)	–	+ (T2)	–	–	–
ZSR	+ (T4)	–	+ (T4)	–	+ (T4)	–	+ (T4)	–	+_(28,159)_	–
MLR	–	–	+ (T4)	–	–	–	–	–	+_(3376)_	–
NGR	–	–	–	–	–	–	–	–	–	–
DJR	–	–	+ (T4)	–	+ (T4)	–	+ (T4)	–	+_(1040)_	–
LYT	–	–	–	+ (T3)	–	+ (T3)	–	+ (T3)	–	+
WS	+ (T4)	–	+ (T4)	+ (T4)	+ (T4)	+ (T4)	+ (T4)	+ (T4)	+_(3132)_	+
SML	+ (T2)	+ (T2)	+ (T2)	+ (T2)	+ (T2)	+ (T2)	+ (T2)	+ (T2)	+_(6008)_	+
TS	–	–	–	–	–	–	+ (T4)	–	+_(783)_	–
MiD	+ (T4)	+ (T4)	+ (T4)	+ (T4)	+ (T4)	+ (T4)	+ (T4)	+ (T4)	+_(15,926,384)_	–
DJ	–	–	+ (T4)	–	+ (T4)	–	+ (T4)	–	–	–
ZWR	–	+ (T5)	+ (T4*)	+ (T5)	+ (T4)	+ (T5)	+ (T4)	+ (T5)	+_(1820)_	+
KPR	–	–	+ (T4)	–	+ (T4)	–	+ (T4)	–	+_(8895)_	–
BNR	–	–	+ (T4)	–	+ (T4)	–	+ (T4)	–	+_(6753)_	–
LT	–	+ (T2)	+ (T2)	+ (T2)	+ (T2)	+ (T2)	+ (T2)	+ (T2)	+_(1820)_	+
RYT	–	–	–	+ (T11)	–	–	–	+ (T11)	–	+
BH	–	–	–	–	–	–	–	–	–	–
WST	–	–	–	+ (T4)	–	–	–	+ (T4)	–	+
AGD	–	–	–	–	–	–	–	–	–	–
FS	+ (T4)	+ (T4)	+ (T4)	+ (T4)	+ (T4)	+ (T4)	+ (T4)	+ (T4)	+_(38,273,385)_	+
CCL	+ (T4)	+ (T4)	+ (T4)	+ (T4)	+ (T4)	+ (T4)	+ (T4)	+ (T4)	+_(731,052)_	+
MuD	–	–	+ (T3)	–	+ (T3)	–	–	–	–	–
Detection rate	37.5%	28.1%	75%	43.8%	65.6%	37.5%	71.9%	43.8%	65.6%	40.6%

Past studies have shown that *Acanthamoeba* spp. have been detected in various aquatic environments worldwide and the presence of *Acanthamoeba* ranges from 3.6 to 73.7% by small volume filtration or culture procedure with Genotyping (JDP) PCR. Most of these reports have shown detection rates ranging from 30 to 50%^2^. The broad range of data may be due to the geographical conditions or diversity of ecological sites around the world. Nevertheless, according to sensitivity and LOD test findings, we suggest that the results of these studies may be underestimated. Other studies based on real-time PCR or nested PCR showed that the presence of *Acanthamoeba* observed from the aquatic environment was higher than Genotyping (JDP) PCR in the same country^7,13,24^. The M3 method used in this study showed a strong impact including the highest detection rates, better sensitivity, and powerful genotyping ability. Therefore, we suggest the use of M3 and real-time PCR (M6) could to find out the actual situation, including genotypes and amount of *Acanthamoeba* in aquatic environments for further risk assessment.

The M3 and M5 were the two sensitive methods used in this study. Some inconsistencies in the detection results of each sample in total PCR-based methods were observed. The results of direct concentration showed that water samples from MLR and MuD sites tested positive using M3, but negative in M5. This difference may be due to the gel resolution of amplicons and the actual LOD of these two methods. However, the DSR water sample was positive in semi-nested PCR and real-time PCR, whereas it was negative in M3. These differences may be due to *Acanthamoeba* genomic DNA damage caused by manual error and the amplicon size of M3 in the first step (outer primer) is approximately 1000 bp. In contrast, M3 had the best sensitivity and could also amplify the ASA.S1 segment of the 18S rRNA gene sequence of *Acanthamoeba*.

Moreover, we carried out *Acanthamoeba* detection by all methods in a river basin and the result was shown in Supplementary Table 2. The detection rate of M3, M4, and M6 were the same (90%), while M5 was 85% and M1 was 10%. This result would support the high occurrence of *Acanthamoeba* in aquatic environments (single sampling site for various rivers and reservoirs or many sampling sites for a river basin). We had carried out the *Acanthamoeba* survey in the same river basin between July 2009 and March 2010 by the M1 method^34^. The detection rate in the past study was 11.7%, it similar to this study (10%) by the same method (M1), however, the real situation for *Acanthamoeba* occurrence was underestimation by the M1 method.

Sensitivity and detection rates of culture-dependent procedure and direct molecular enumeration procedure for detecting *Acanthamoeba* in the aquatic environment are contradictory. Surprisingly, few reports have shown that the culture procedure is more sensitive than the direct concentration procedure while others have opposed the same^39,44–46^. Our results provide a reasonable explanation to support that the direct concentration procedure is usually sensitive than the culture-dependent procedure explaining the advantages and disadvantages of both methodologies. The advantages of direct concentration procedure include (1) easy to harvest *Acanthamoeba* and (2) high sensitivity of much lower LOD (Limitation of Detection) by molecular methods, e.g. real-time PCR, nested PCR and LAMP; in contrast, the disadvantages include the presence of molecular inhibitor from water and in case lower concentration density. The growth incubation step provides an advantage of the culture-dependent procedure. However, its culturing method is very challenging which could results in the generation of more cysts since *Acanthamoeba* grows very slowly, and can easily overgrow by other environmental organisms such as bacteria, fungi, or other amoebae. Cysts are more environmentally resistant and may not easily break down by lysis buffer failing to extract DNA. Furthermore, the water body must be shaken violently made uniform before filtering the water body, which may also damage the *Acanthamoeba*^2^, leading to failure of the culture-dependent procedure. Hence, the detection rate and sensitivity of the direct molecular enumeration procedure were found better due to higher sensitivities. In summary, the presence of *Acanthamoeba* in the aquatic environment in Taiwan was higher, resulting in 87.5% positivity with almost all the methods. The study regarding free-live amoeba in Spain indicated that *Acanthamoeba* is the commonly found genus in the various aquatic environments and has shown higher detection rate, 99.1% in 223 water samples^13^.

The high occurrence of T4 genotype *Acanthamoeba* (75%) from rivers and reservoirs in this study poses an important issue for public health since the T4 genotype, out of the currently recognized 20 genotypes, is the most common cause of keratitis-inducing *Acanthamoeba*^12^. A systematic study for *Acanthamoeba* conducted in a total of 427 environmental isolations showed that the genotypes T4, T3, T5, and T2 accounted for 48%, 13%, 13%, and 11% of the total detected isolates, respectively^47^. The genotyping results in this study agreed with the systematic analysis study and our previous study at the same sampling site showing that T4, T3, T5, and T2 genotypes were the most common. The T4 and T3 genotypes were mostly detected in AK patients, whereas the T4, T1, and T2 genotypes were mostly detected in GAE patients^47^. The pathogenic risk of *Acanthamoeba* in Taiwan is prevalent; therefore, extensive initiatives, such as the current investigation, are supported by the Taiwan Centers for Disease Control (MOHW105-CDC-C-114-122109). The ultimate aim of this research and prevalence studies is to establish information on the distribution and risk factors of important water-borne protozoan parasites that can be used as a reference for future policymaking and outbreak response strategies. This study provides a useful method for detecting *Acanthamoeba* in aquatic environments, and suggests that public health agencies require long-term surveys, especially under climate change threats^49^. Novel disinfection strategies with active molecules and enzymes can also be evaluated for their inhibition efficiency using the current method^50^. Moreover, the high prevalence of the *Acanthamoeba* T4 genotype in aquatic environments indicates a potential threat to public health. According to the history of patients diagnosed with AK and GAE, wearing contact lenses and contact with recreational/agriculture water were the main risk factors, while soil/dust as a source of infection and the effect of climate change require a thorough investigation^48–52^. Overall, along with the detection of contamination sources and accurate estimation, better hygiene, implementation of disinfection methods, and pathogen safety measures are warranted to avoid the risk of *Acanthamoeba* infection.

The ZWR water sample showed different genotyping results in the direct concentration and Culture procedures, T4 and T5 genotypes were detected respectively in the two procedures. To explain this, we used a DGGE assay to analyse the amplicons from M3. The DGGE result showed the contamination of both the T4 and T5 genotypes in the water sample. Our previous study had shown T3, T4 and T5 genotypes mixed contamination in the ZWR sampling site in different sampling research in the past^8^. These results explain the long-term genotypes mixing and contamination in the ZWR sampling site and the predominance of T4 and T5 genotypes. Therefore, using the M3 combined with DGGE assay could easily characterize these genotypes mixing and contamination in the aquatic environment.

In a previous study from Taiwan, *Acanthamoeba* spp. in cooling tower water and biofilm samples were reported in the range of 2 × 10^3^–3 × 10^6^ and 1.3 × 10^3^–8.4 × 10^5^ copies/L, respectively^5^. In Germany and Taiwan, *Acanthamoeba* spp. in groundwater samples (River and Reservoirs) were detected in the range of 1.2 × 10^3^–5.4 × 10^6^ and 1.8 × 10^3^–1.1 × 10^5^ copies/L, respectively^53,54^. The results of this study in quantifying *Acanthamoeba* spp. in river water or groundwater are at par with the previous studies. Empirical test results provided a good explanation as to why using a small volume for collection is enough. The detection range by real-time PCR assay is approximately 1 × 10^3^ copies/L in various aquatic environments worldwide. According to our LOD test result, the LOD of our nested PCR, semi-nested PCR, and real-time PCR are approximately 50 copies/reaction or lower. Therefore, collecting a 1 L water sample followed by filtration, genomic DNA extraction to 100 μL and further carrying out molecular assays using 5 μL DNA with highly sensitive PCR methods is enough for surveillance purposes. According to empirical test results (Table 3), the LOD of M3 is approximately 35 copies/reaction.

*Acanthamoeba* is ubiquitously found in various aquatic environments, suggesting that it may play an important ecological role. One of the most important roles of FLA is that it acts as a host for several human pathogens, such as enterovirus, norovirus, coxsackievirus, adenovirus, *Mycobacterium avium*, *Campylobacter*, *Legionella* spp., *Streptococcus pneumoniae*, *Streptococcus pyogenes,* and *Listeria* spp.^3,4,14,55–61^. Among these, the survival of *enterovirus*, *rotavirus, norovirus*, *coxsackievirus*, *S. pneumoniae,* and *S. pyogenes* was commonly reported in the genus *Acanthamoeba*. *Acanthamoeba* can be highly resistant to disinfectants, which can allow bacterial or viral viability in the environment and can account for poorer disinfection of water, especially virus-*Acanthamoeba* interactions. The occurrence of human infectious viruses and bacteria within amoebae is a public health concern, urging the need to carry out further studies on amoeba-resistant bacteria from the natural environment.

It is well known that the presence of free-living amoebae poses a potential public health challenge. Quantitative real-time PCR combined with a sensitive genotyping method may significantly contribute to epidemiological knowledge about the genotype and abundance of *Acanthamoeba* spp. in aquatic environments by establishing quantitative microbial risk assessment (QMRA) in future.

## Conclusions

1. The current study established a highly sensitive genotyping method for detecting *Acanthamoeba* spp. in water samples, which requires a small sample volume. Determination of the detection limits, in silico tests, and empirical tests for *Acanthamoeba* spp. were performed by comparing various PCR-based methods.
2. It is noteworthy that this study showed a high prevalence of *Acanthamoeba* spp. in aquatic environments in Taiwan. This result is corroborated by previous studies suggesting that *Acanthamoeba* spp. are one of the most commonly found free-living amoeba in natural aquatic environments, and their prevalence might be underestimated due to the use of the single PCR method.
3. The T4 genotype is the most common in the aquatic environment of Taiwan which is supported by previous observations on *Acanthamoeba* genotyping.
4. A possible explanation for the difference between culture-dependent and direct determination by the molecular procedure was underpinned in this study, and we found evidence that suggests the direct enumeration procedure combined with nested PCR method in field study is the most efficient approach. Hence, we suggest that M3 combined with real-time PCR is the best genotyping and quantitative method.
5. To our knowledge, this is the first report comparing the primer annealing efficiency of various PCR-based methods which also provided substantial evidence that M3 is the most sensitive method based on comparison with control and empirical tests.

## Supplementary Information

Supplementary Table 1.

Supplementary Table 2.

## Data Availability

All sequencing data, figures and tables of this study have been placed in website Figshare. (https://figshare.com/s/ff4fa72321b08b3e86d9). All sequence data from the samples has been submitted to GenBank (at www.ncbi.nlm.nih.gov) and the assigned accession numbers were from MK390840 to MK390877.
