# Supplementary material for: Efficient nested-PCR-based method development for detection and genotype identification of Acanthamoeba from a small volume of aquatic environmental sample
Source: Sci Rep. 2021 Nov 5;11:21740. doi: 10.1038/s41598-021-00968-2 (PMC8571327; doi:10.1038/s41598-021-00968-2)
Supplement: Supplementary file 1 — Supplementary Table 1. [file 41598_2021_968_MOESM1_ESM.pdf]

Supplementary Table 1. Methods for calculating sensitivity/specificity/accuracy from empirical test based on sequencing positive sample.

Methods	Sensitivity			Specificity			Accuracy		
	D.C	Culture	Total	D.C	Culture	Total	D.C	Culture	Total
**Genotyping PCR (M1)**	43% (12/28)	32% (9/28)	50% (14/28)	100% (4/4)	100% (4/4)	100% (4/4)	50% (16/32)	41% (13/32)	56% (18/32)
**Optimal modified Genotyping Nested PCR (M3)**	86% (24/28)	50% (14/28)	96% (27/28)	100% (4/4)	100% (4/4)	100% (4/4)	88% (28/32)	56% (18/32)	97% (31/32)
**Scheikl Genotyping Nested PCR (M4)**	75% (21/28)	43% (12/28)	79% (22/28)	100% (4/4)	100% (4/4)	100% (4/4)	78% (25/32)	50% (16/32)	81% (26/32)
**Genotyping Semi-nested PCR (M5)**	82% (23/28)	50% (14/28)	93% (26/28)	100% (4/4)	100% (4/4)	100% (4/4)	84% (27/32)	56% (18/32)	94% (30/32)
**Qvarnstrom Real-time PCR (M6)**	75% (21/28)	46% (13/28)	86% (24/28)	100% (4/4)	100% (4/4)	100% (4/4)	78% (25/32)	53% (17/32)	88% (28/32)
