# Supplementary material for: Efficient nested-PCR-based method development for detection and genotype identification of Acanthamoeba from a small volume of aquatic environmental sample
Source: Sci Rep. 2021 Nov 5;11:21740. doi: 10.1038/s41598-021-00968-2 (PMC8571327; doi:10.1038/s41598-021-00968-2)
Supplement: Supplementary file 2 — Supplementary Table 2. [file 41598_2021_968_MOESM2_ESM.pdf]

Supplementary Table 2. Summary of different PCR methods used to detect *Acanthamoeba* in Puzih River

**Sampling locations**	**Genotyping PCR (M1)**<br>JDP	**Modify Genotyping Nested PCR (M3)**<br>ComFLA →F900+JDP2-M	**Nested PCR (M4)**<br>JDP1+P3rev →P2fw+JDP2	**Semi-Nested PCR (M5)**<br>JDP →A1+JDP2	**Real-time PCR (M6)**<br>AcanF900+AcanP1000 +AcanR1100
**2016Q2 PR 1**	+	+	+	+	+
**2016Q2 PR 3**	+	+	+	+	+
**2016Q2 PR 5**	-	+	+	+	+
**2016Q2 PR 7**	-	+	+	+	+
**2016Q2 PR 8**	-	+	+	+	+
**2016Q2 PR 9**	-	+	+	+	+
**2016Q2 PR 10**	-	+	+	+	+
**2016Q2 PR 12**	-	+	+	+	+
**2016Q2 PR 14**	-	+	+	+	+
**2016Q2 PR 16**	-	+	+	+	+
**2016Q2 PR 18**	-	+	+	+	+
**2016Q2 PR 20**	-	+	+	+	+
**2016Q2 PR 21**	-	+	+	+	+
**2016Q2 PR 22**	-	+	+	+	+
**2016Q2 PR 23**	-	+	+	+	+
**2016Q2 PR 25**	-	+	+	+	+
**2016Q2 PR 30**	-	-	-	-	-
**2016Q2 PR 31**	-	+	+	-	+
**2016Q2 PR 32**	-	+	+	+	+
**2016Q2 PR 34**	-	-	-	-	-
**Detection rate**	10%	90%	90%	85%	90%
